# Optimization of Specific Productivity for Xylonic Acid Production by *Gluconobacter oxydans* Using Response Surface Methodology

**DOI:** 10.3389/fbioe.2021.729988

**Published:** 2021-08-13

**Authors:** Tao He, Chaozhong Xu, Chenrong Ding, Xu Liu, Xiaoli Gu

**Affiliations:** ^1^ Jiangsu Co-Innovation Center of Efficient Processing and Utilization of Forest Resources, College of Chemical Engineering, Nanjing Forestry University, Nanjing, China; ^2^ Jiangsu Key Lab of Biomass-based Green Fuels and Chemicals, Nanjing, China

**Keywords:** xylonic acid, specific productivity, oxygen transfer, oxygen uptake, response surface methodology

## Abstract

Large amounts of xylose cannot be efficiently metabolized and fermented due to strain limitations in lignocellulosic biorefinery. The conversion of xylose into high value chemicals can help to reduce the cost of commercialization. Therefore, xylonic acid with potential value in the construction industry offers a valuable alternative for xylose biorefinery. However, low productivity is the main challenge for xylonic acid fermentation. This study investigated the effect of three reaction parameters (agitation, aeration, and biomass concentration) on xylose acid production and optimized the key process parameters using response surface methodology The second order polynomial model was able to fit the experimental data by using multiple regression analysis. The maximum specific productivity was achieved with a value of 6.64 ± 0.20 g g_x_^−1^ h^−1^ at the optimal process parameters (agitation speed 728 rpm, aeration rate 7 L min^−1^, and biomass concentration 1.11 g L^−1^). These results may help to improve the production efficiency during xylose acid biotransformation from xylose.

## Introduction

Xylose, which constitutes about 25% of the total biomass components, is unable to be converted in the lignocellulosic biorefinery process due to the limitation of microorganisms (Zaldivar et al., 2001). Therefore, the utilization of xylose is one of the key factors affecting the commercial production of lignocellulose (Nogue and Karhumaa, 2015). Currently, the bioconversion of xylose to xylonic acid is considered as a promising pathway and has gained much attention (Zhou et al., 2015, Zhou et al., 2017). Xylonic acid (XA), a bio-based chemical of great interest in recent years, is a non-toxic, non-volatile, non-corrosive, water-soluble organic acid. This aldonic acid compound has a similar structure and properties to gluconic acid, and shows a wide variety of applications in various fields. XA can be used as a dispersant to improve the dispersibility of concrete, which can effectively reduce the amount of concrete. In addition, XA has been also used as a raw material for chelating agent, antibiotic, polyamide, hydrogel modifier and 1,2,4-butanetriol precursor (Gupta et al., 2001; Deppenmeier et al., 2002; Chun et al., 2006).

Compared to other bacteria such as *Saccharomyces cerevisiae* and *Escherichia coli, Gluconobacter oxydans* has been the most productive in producing XA (Toivari et al., 2012a). *G. oxydans* is known for its rapid but incomplete oxidation of sugars and sugar alcohols as an obligate aerobic bacterium with a wide range of industrial applications (Toivari et al., 2012b; Zhang et al., 2016a). XA is derived primarily from the further oxidation of xylose, relying on xylose dehydrogenase on the cell membrane of *G. oxydans* (Mientus et al., 2017). Then, XA is recovered from the culture broth by ion exchange or precipitation methods for downstream processing (Liu et al., 2012; Cao and Xu, 2019). Although *G. oxydans* shows great potential for XA fermentation, low production efficiency is the main challenge. For bioengineering, an important goal is to achieve high productivity in order to ensure an adequate supply of valued product and to reduce manufacturing costs (Handlogten et al., 2018). Therefore, improving the production efficiency of *G. oxydans* oxidized xylose would provide new options for large-scale production of XA.

For aerobic bacteria, the inability to utilize sufficient oxygen is the main cause of low productivity. Biochemically speaking, the xylose biocatalysis by *G. oxydans* is a closely coupled bio-oxidation reaction of cellular respiration and dehydrogenation that relies heavily on oxygen supply (Hoelscher et al., 2009). However, the solubility of oxygen in the broth is less than 0.21 mmol L^−1^, and the metabolism of bacterial cells is significantly constrained (Hua et al., 2020), leading to a decrease in the catalytic performance of the biological process (Garcia-Ochoa and Gomez, 2009). Therefore, the level of oxygen utilization is a key factor in the final production efficiency.

Often, oxygen utilization is related to the interaction between the oxygen transfer and the oxygen uptake. During aerobic biological reactions, oxygen transfer is the process by which oxygen is transferred from the gas into the liquid phase to come into contact with the cells, while oxygen uptake is the process by which oxygen is utilized by the cells (Garcia-Ochoa et al., 2010). Theoretically, increasing the biomass concentration would enhance the level of oxygen uptake for aerobic biological reactions, but it would break the relationship between oxygen uptake and oxygen transfer. This leads to a decrease in the dissolved oxygen concentration in the broth and thus reduces the catalytic performance of the bacteria, which ultimately leads to a decrease in productivity. Therefore, it is necessary to adjust the appropriate level of oxygen transfer (by changing the agitation and aeration) and oxygen uptake (by changing the biomass concentration) to increase productivity.

To address these issues, this study attempts to use a response surface approach to improve the productivity of xylonic acid fermentation. Response surfaces were used to understand the interactions of aeration, agitation, and biomass concentration on the productivity of xylonic acid production from whole-cell catalytic xylose by *G. oxydans* using a statistical model. Optimized fermentation conditions were designed to address the low productivity limitations that exist in xylonic acid fermentation. These optimized conditions will provide guidance for the design of xylonic acid production process.

## Materials and Methods

### Microorganism

*G.oxydans* NL71 purchased from Nanjing Forestry University of China, was stored on sorbitol agar (sorbitol 50 g L^−1^, yeast extract 5 g L^−1^, agar 15 g L^−1^) at 4°C. The inoculum of *G. oxydans* NL71 was prepared in 250 ml shake flasks containing 50 ml of medium (sorbitol 50 g L^−1^, yeast extract 5 g L^−1^) and incubated at 220 rpm and 30°C for 24 h. The proliferation medium was centrifuged at 6,000 *g* for 5–10 min to collect cells, and the centrifuged cells were transferred to the fermentation medium on an ultra-clean bench (Zhou et al., 2015).

### Whole-Cell Catalysis

The fermentation equipment consists of a stirred tank bioreactor, motor, and control system. Whole-cell catalysis is performed in a 5 L stirred tank reactor containing 3 L of production medium. The biotransformation medium: 5.0 g L^−1^ yeast extract, 0.5 g L^−1^ MgSO4, 1.0 g L^−1^ KH_2_PO_4_, 2.0 g L^−1^ K_2_HPO_4_ and 5.0 g L^−1^ (NH_4_)_2_SO_4_ (Zhou et al., 2017). The temperature was maintained at 30°C throughout the fermentation process. The pH of the medium was monitored online by means of electrodes connected to the pH meter. The peristaltic pump was controlled to add 10% NaOH to maintain the pH at 5.5 automatically (Zhou et al., 2015; Zhang et al., 2017; Zhou et al., 2017).

### Experimental Design and Statistical Optimization

A three-factor, three-level Box-Behnken experiment design was used, with specific productivity values (*Px*) as response values to understand the combined effect of agitation (A), aeration (B) and biomass concentration (C). Table 1 shows the range of agitation, aeration, and biomass concentration of the RSM.

**TABLE 1:** Experimental range of variables studied during designing of experiments.

Factors	Symbols and units	Coded levels		
		Low (-1)	Mid (0)	High (+1)
Agitation	A (rpm)	200	500	800
Aeration	B (L min^−1^)	1	4	7
Biomass concentration	C (g L^−1^)	1	1.5	2

Fifteen experiments (Table 2) were designed using Design-Expert 12, and the fermentation results were finally subjected to multiple regression analysis to obtain the optimal fermentation conditions. A second-order polynomial equation was designed to express the predicted response (*Y*) as a polynomial equation in the independent variable (*A*-*C*), expressed as Eq. 1:

$$Y=x_{0}+x_{1}A+x_{2}B+x_{3}C+x_{11}A^{2}+x_{22}B^{2}+x_{33}C^{2}+x_{12}\mathrm{AB}+x_{13}\mathrm{AC}+x_{23}\mathrm{BC}$$ (1)

where *Y* is the response, $x_{0}$is the intercept coefficient,$x_{1}$,$x_{2}$, $x_{3}$are the linear coefficients and$x_{11}$,$x_{22}$,$x_{33}$ are the squared coefficients; and$x_{12}$, $x_{13}$, and$x_{23}$ are the interaction coefficients.

**TABLE 2:** Experimental matrix design.

Run	Factor A	Factor B	Factor C	Response: *P*_*X*_
	Agitation (rpm)	Aeration (L min^−1^)	Biomass concentration (g L^−1^)	(g g_x_^−1^ h^−1^)
1	200	1	1	0.32
2	200	7	1	1.28
3	500	4	1	5.75
4	800	1	1	6.00
5	800	7	1	6.33
6	200	4	1.5	0.94
7	500	1	1.5	4.67
8	500	4	1.5	5.50
9	500	7	1.5	5.76
10	800	4	1.5	6.01
11	200	1	2	0.33
12	200	7	2	0.93
13	500	4	2	3.18
14	800	1	2	4.07
15	800	7	2	4.45

### Biomass Measurement

The optical density (OD) of the cells was measured at 600 nm using a UV-Vis spectrophotometer (Ultrospec 2100, Amersham Biosciences Corp., United States). Samples were diluted with deionized water and a blank control was also used for the measurements.

### Determination of Xylose and Xylonic Acid Content

The simultaneous determination of xylose and XA was performed using high performance anion exchange chromatography combined with pulsed amperometric detection (Thermo ICS-5000) using a CarboPac™ PA10 column (Wang et al., 2014). The samples were filtered (0.22 μm membrane) and injected (10 μL) on a CarboPac PA-10 guard column (2 mm × 50 mm) attached to a CarboPac PA-10 anion-exchange analytical column (2 mm × 250 mm). The column temperature was maintained at 30°C. 100 mM NaOH was used as mobile phase at a flow rate of 0.3 ml min^−1^. Before each injection, the column was re-equilibrated by running for 15 min with 6 mM NaOH to achieve good repeatability.

### Specific Productivity and Volumetric Productivity

XA specific productivity (*P*_*X*_) was calculated for each experiment employing Eq. 2:

$$P_{X}=\frac{C_{XA}^{\max}}{C_{X}\cdot t^{\max}}$$ (2)

Volumetric productivity (*Q*_*P*_) was calculated by Eq. 3:

$$Q_{P}=\frac{C_{XA}^{\max}}{t^{\max}}$$ (3)

where $C_{XA}^{\max}$ (g L^−1^) is the maximum XA concentration for each experiment, *C*_*X*_ is the biomass concentration (g L^−1^) and *t* ^*max*^ (h) is the time in which the maximum XA concentration is reached.

### Determination of *k*_*L*_ *a*

The dissolved oxygen concentration was monitored using a fast dissolved oxygen electrode (605-ISM, Mettler Toledo, United States), and the *k*_*L*_ *a* values were determined by the dynamic method. First, aeration was stopped, and dissolved oxygen levels were decreased to measure oxygen uptake rate. Then, the broth was reaerated with air until the steady state was reached, and *k*_*L*_ *a* value was calculated by the dissolved oxygen mass balance equation (Garcia-Ochoa and Gomez, 2009).

## Results and Discussion

### Statistical Analysis

In comparison to univariate experiments, RSM takes into account the interaction of all factors on response variables, and also obtains appropriate optimization conditions. In this study, the RSM examined the effect of three variables: agitation (A), aeration (B) and biomass concentration (C) on *P*_*X*_. The evaluation results show that the model has 9 degrees of freedom (df) and the ‘lack of fit’ has 5 df, indicating that the design is suitable for model development. The model with second-order polynomial was defined according to the coding factor as follows:

$$\begin{array}{l} Y=5.21+2.31A+0.3366B-0.6698C-0.1057\mathrm{AB}-0.4338\mathrm{AC}-0.0386\mathrm{BC}-1.67A^{2}\\ +0.0725B^{2}-0.6721C^{2} \end{array}$$ (4)

The results in Table 3 were verified by Fisher’s test for analysis of variance (ANOVA), which showed that the experimental data fitted well with the second-order polynomial function. The Model F-value of 44.44 implied the model was significant. The probability of not getting the results predicted by the regression model is only 0.01%. The large *p*-value for ‘lack of fit’ (0.1273 > 0.05) indicated that the “lack of fit” is not significant relative to the pure error. The *R* ^2^ value of 98.28% indicated that the response model explains 98.28% of the total variation. Usually, regression models with *R* ^2^ values above 0.9 are considered to have a robust correlation (Ávila-Lara et al., 2015). The value of the adjusted coefficient of determination (R^2^_Adj_ = 96.07%) was also high enough to indicate the significance of the model. These results all suggest that the model can appropriately account for the cumulative as well as univariate effects of all selected variables on productivity in this biotransformation system.

**TABLE 3:** Analysis of variance (ANOVA) for all model terms.

Source	Sum of squares	df	Mean square	F-value	*p*-value
Model	77.82	9	8.65	44.44	<0.0001
A-Agitation	53.27	1	53.27	273.77	<0.0001
B-Aeration	1.13	1	1.13	5.80	0.0469
C-Biomass concentration	4.49	1	4.49	23.06	0.0020
AB	0.0894	1	0.0894	0.4597	0.5196
AC	1.50	1	1.50	7.71	0.0274
BC	0.0114	1	0.0114	0.0588	0.8154
A^2^	7.44	1	7.44	38.21	0.0005
B^2^	0.0141	1	0.0141	0.0723	0.7957
C^2^	1.21	1	1.21	6.22	0.0413
Residual	1.36	7	0.1946	—	—
Lack of Fit	1.29	5	0.2580	7.14	0.1273
Pure Error	0.0722	2	0.0361	—	—
Cor Total	79.18	16	—	—	—

**FIGURE 1:**
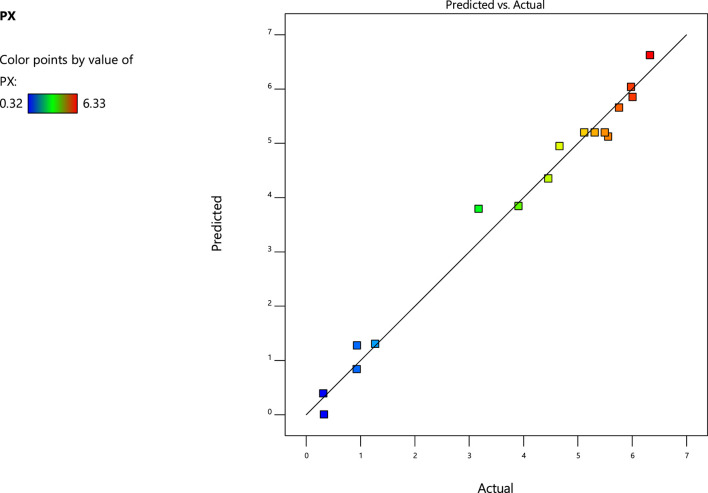
Parity plot: showing the relation between actual response and predicted values for *P*_*X*_ estimation.

Figure 1 shows the good correlation between the predicted values and the actual values. The predicted values of the model can match well with the actual values, indicating the good applicability of the model. The model gave the optimized levels of agitation speed (728 rpm), aeration rate (7 L min^−1^), and biomass concentration (1.11 g L^−1^), and experiments were conducted in the fermenter. The final *P*_*X*_ reached 6.64 ± 0.20 g g_x_^−1^ h^−1^, which is slightly lower than the model value of 6.74 g g_x_^−1^ h^−1^.

### Interactive Effect of the Selected Variables on *P*_*X*_

**FIGURE 2:**
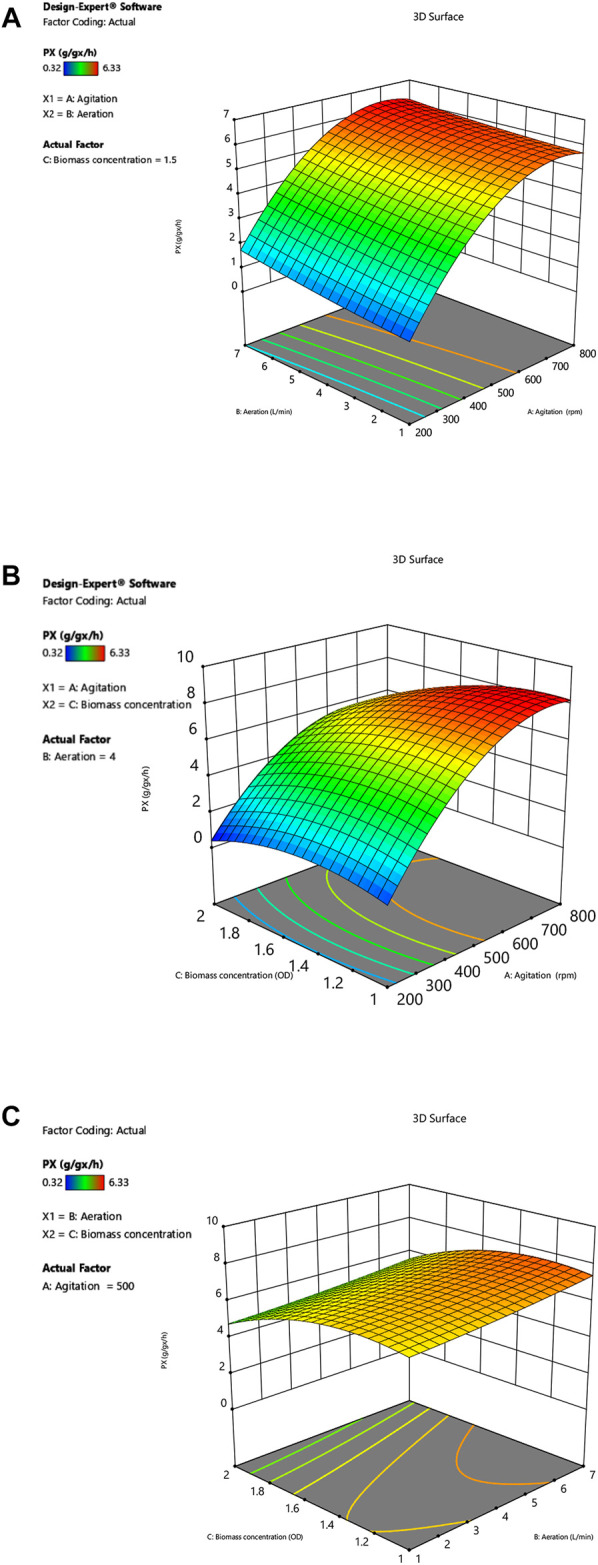
**(A)** Interactive effect of agitation and aeration on *P*_*X*_. **(B)** Interactive effect of agitation and biomass concentration on *P*_*X*_. **(C)** Interactive effect of aeration and biomass concentration on *P*_*X*_.

**FIGURE 3:**
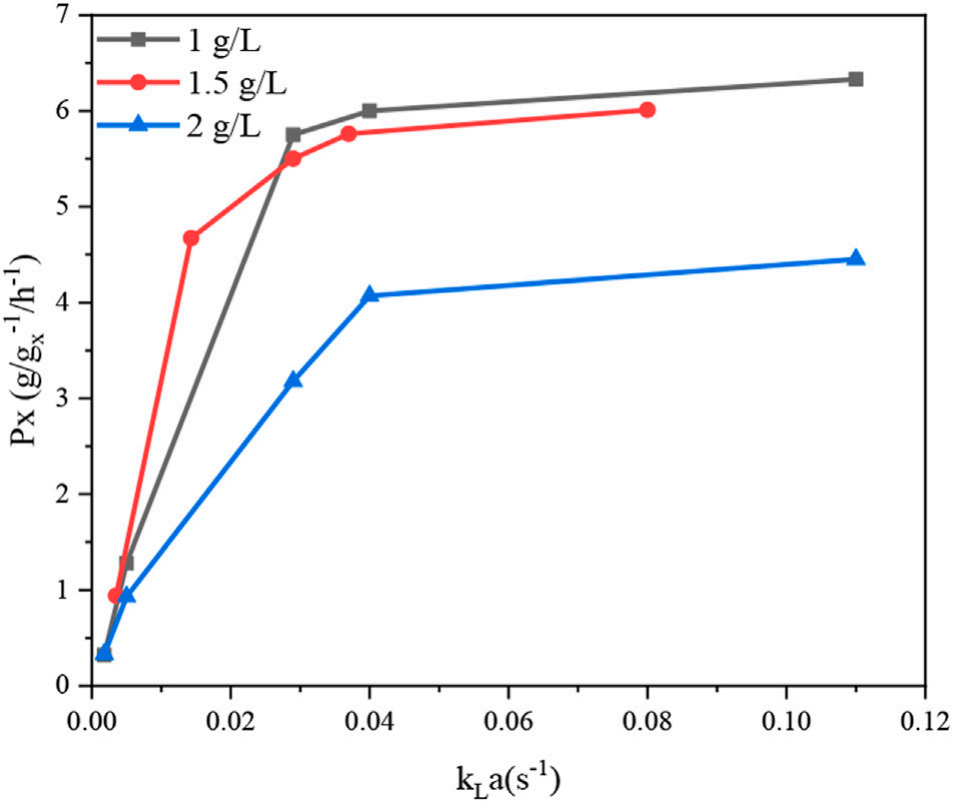
Specific productivity of XA production using different concentrations of *G. oxydans* NL71 at different *k*_*L*_ *a* conditions.

Three different response surface plots (Figures 2A–C) were used to determine the interaction effect between the selected variables on *P*_*X*_. In Figure 2A, the response surface plot shows that the *P*_*X*_ value was small at lower agitation speed and aeration rates; as the value of agitation and aeration increase, *P*_*X*_ was also enhanced. Theoretically, both agitation speed and aeration affect the oxygen transfer in the fermenter, and an increase in the agitation speed and aeration rates can effectively increase the oxygen transfer coefficient (*k*_*L*_ *a*) in the fermenter (Table 4). Meanwhile, Figure 3 shows that *P*_*X*_ was significantly improved as *k*_*L*_ *a* was increased from 0.0018 s^−1^ to 0.04 s^−1^, while the increase in *P*_*X*_ flattened out as *k*_*L*_ *a* continued to increase. In addition, similar productivity was achieved when *k*_*L*_ *a* was increased from 0.04 s^−1^ to 0.08 s^−1^ at a biomass concentration of 1 g L^−1^ and 1.5 g L^−1^, respectively. From these results, it can be concluded that the production efficiency of *G.oxydans* can be effectively increased with an appropriate value of *k*_*L*_ *a*. However, the production efficiency could not be further improved by increasing *k*_*L*_ *a* to a higher level. It can be considered that the overall rate of the biological process is controlled by oxygen transfer when *k*_*L*_ *a* is less than 0.04 s^−1^. When increasing the oxygen transfer coefficient to more than 0.04 s^−1^, the reaction efficiency cannot be further improved effectively. At this stage, the overall rate of the biological process is controlled by oxygen uptake. In addition, it can be observed that the curve of aeration is slightly curved on the surface. This observation suggests that the dominant factor in the interaction is agitation, which has been confirmed by previous studies (Dixit et al., 2015; Li et al., 2019). This is in agreement with the report of Pooja Dixit et al. (2015), who also found that agitation has the most decisive effect on *k*_*L*_ *a*. The reason is that agitation reduces the bubble size and increases the gas-liquid contact area.

**TABLE 4:** Mass transfer coefficients (*k*_*L*_ *a*) in fermenters under different operating conditions.

Run	Agitation (rpm)	Aeration (L min^−1^)	*k*_*L*_*a* (s^−1^)
1	200	1	0.0018
6	200	4	0.0035
2	200	7	0.005
7	500	1	0.0143
3	500	4	0.029
9	500	7	0.037
4	800	1	0.04
10	800	4	0.08
5	800	7	0.11

However, it can be observed that the *P*_*X*_ value tends to a plateau in the high agitation region (Figure 2A). In addition, an examination of the data (Table 2) showed that the increase in agitation value from 200 to 500 rpm (run no. 6 and 8) caused an increase in *P*_*X*_ value from 0.94 g g_x_^−1^ h^−1^–5.50 g g_x_^−1^ h^−1^, whereas an increase in agitation from 500 to 800 rpm (run no. 8 and 10) increased the *P*_*X*_ value from 5.50 g g_x_^−1^ h^−1^–6.01 g g_x_^−1^ h^−1^, which was not so significant as compared to previous experiments (run no. 6 and 8). It can be concluded that the improved oxygen transfer conditions brought about by the agitation speed of 800 rpm provide an adequate oxygen supply for oxygen uptake, thus enabling the maximum catalytic performance of the strain.

Figure 2B shows the dependence of *P*_*X*_ on biomass concentration *C*_*X*_ and agitation at a specific aeration rate. In the low agitation region, the value of *P*_*X*_ is small; as the agitation speed is enhanced, *P*_*X*_ is also increased. The *P*_*X*_ increase tends to level off when the agitation speed exceeds 500 rpm. The maximum *Px* of 6.33 g g_x_^−1^ h^−1^ was reached at an agitation speed of 800 rpm, which indicates that the catalytic capacity of the bacteria is close to the maximum value at this operating condition. When the capacity of oxygen uptake was enhanced, the results showed that *P*_*X*_ instead decreased. It can be confirmed by comparing the data of run 3 and 13 (Table 2), where *P*_*X*_ decreased from 5.75 g g_x_^−1^ h^−1^–3.18 g g_x_^−1^ h^−1^ by increasing the biomass concentration from 1 g L^−1^–2 g L^−1^. Previously, Yuan et al. (2016) reported similar observations and suggested that the oxygen limitation brought about by the growth of the bacterium at the beginning of fermentation could affect the expression of transhydrogenase on the *G. oxydans* membrane, which might reduce the catalytic performance of the bacteria. Theoretically, an increase in biomass concentration could improve the level of oxygen uptake, but the imbalance between oxygen uptake and oxygen transfer leads to lower dissolved oxygen levels in the medium. Lack of oxygen may affect the activity of enzymes on the cell membrane.

Figure 2C depicts the effect of biomass concentration and agitation on *P*_*X*_ at a specific agitation rate. The response surface plots show that the interaction between aeration rate and biomass concentration on *P*_*X*_ is not significant. Although *Px* is appropriately enhanced by increasing aeration rate, simultaneous increase of in aeration and biomass concentration cause a decrease in *P*_*X*_. The reason is that the increase in biomass concentration leads to an increase in oxygen demand by the cells, while the increase in aeration rate does not bring about optimal oxygen transfer conditions, resulting in oxygen transfer limitation.

### Optimization of Specific Productivity for Xylonic Acid Fermentation

**FIGURE 4:**
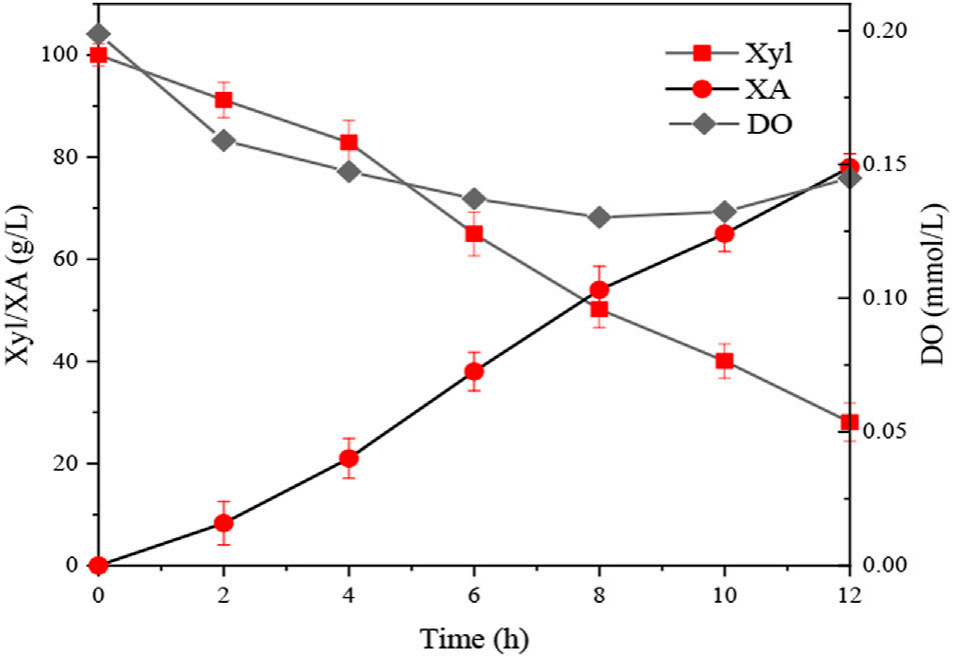
XA production under the optimized conditions. Xyl = xylose; XA = xylonic acid; DO = dissolved oxygen.

By solving Eq. 4, the optimal operating conditions were estimated to be agitation speed 728 rpm, aeration rate 7 L min^−1^, and biomass concentration 1.11 g L^−1^ by response surface analysis. Triplicate fermentation experiments were conducted using the predicted optimal conditions to verify the applicability of Eq. 4. After 12 h of whole-cell catalysis, the DO, xylose, and XA content of the fermentation broth decreased from 0.200 mmol L^−1^ to 0.140 mmol L^−1^, 100 g L^−1^ to 21.04 ± 2.6 g L^−1^, and 0 g L^−1^ increased to 88.44 ± 2.7 g L^−1^, respectively (Figure 4).The experimental value of *P*_*X*_ was 6.64 ± 0.20 g g_x_^−1^ h^−1^, which is in excellent agreement with the predicted value (6.74 g g_x_^−1^ h^−1^).

### A Comparison of Specific Productivity for Xylonic Acid Fermentation in the Literature

It can be said that the commercialization of any fermentation technology is impossible without the economic cost considerations. Therefore, it is important to produce xylonic acid economically with a high volumetric productivity. In the literature, many strategies have been conducted to obtain high product titer of xylonic acid. For example, Toivari et al. (2012a) reported that *S. cerevisiae* was engineered to increase xylonic acid production by genetic engineering. 43 g L^−1^ of xylonic acid was produced in *S. cerevisiae B67002 xylB* with a specific productivity of 0.06 g g_x_^−1^ h^−1^. However, the much lower productivity compared to *G. oxydans* is still a serious weakness. Hahn et al. (2020) reported the effect of different nitrogen sources on the growth and subsequent xylonic acid production. With the addition of 0.32 g L^−1^ glutamate and 0.15 g L^−1^ ammonium sulfate as inexpensive nitrogen sources, a xylonic acid volumetric productivity of 2.92 g L^−1^ h^−1^ is obtained. The increase in the volumetric productivity was due to the higher cell content obtained by changing the medium conditions. However, the volumetric productivity of 2.92 g L^−1^ h^−1^ is still lower when compared to other literature. In shake flask fermentation, oxygen utilization is not the main issue. However, oxygen transfer limitations should be considered when the fermentation system changes from a shake flask to a fermenter. Table 5 shows a comparison of the experimental results for xylonic acid production by other authors. Dai et al. (2020) reported an attempt to use powdered activated carbon treatment to reduce the viscosity of concentrated pre-hydrolysis products and other non-sugar compounds, and 143.6 g L^−1^ XA and *Qp* of 4.48 g L^−1^ h^−1^ were achieved at 300 rpm. Reducing the viscosity of the medium can change the physicochemical properties and improve the oxygen transfer coefficient, but the final *Px* of the biotransformation process was only 1.12 g g_x_^−1^ h^−1^.The result indicating that this mass transfer conditions was not sufficient to maximize the catalytic capacity of the strain. Zhang et al. (2017) reported the production of XA using xylose from cellulosic ethanol distillation distillate with a Qp of 5.42 g L^−1^ h^−1^ at 500 rpm and 2.5 L min^−1^. This study provided a practical process option for the production of XA from lignocellulosic feedstock, but the Px was only 2.18 g g_x_^−1^ h^−1^. The agitation rate at 500 rpm and aeration rate at 2.5 L min^−1^ still does not take advantage of the maximum catalytic capacity of this strain when compared to other reports in the literature. Zhou et al. (2017) reported the improvement of oxygen transfer by increasing the fermenter pressure and introducing pure oxygen, and obtain a total *Qp* of 32.5 g L^−1^ h^−1^ and a *P*_*X*_ of 5.26 g g_x_^−1^ h^−1^ at a biomass concentration of 6.08 g L^−1^, which is the maximum volumetric yield and specific productivity achieved so far. The elevated pressure changes the solubility of oxygen in the medium and the supply of pure oxygen increases the dissolved oxygen concentration, resulting in a significant improvement in oxygen delivery. The high level of oxygen supply makes it feasible to provide the large amounts of oxygen required for high biomass concentration, thus achieving high productivity. However, the costs associated with the supply of pure oxygen and the potential operational difficulties under elevated pressure conditions cannot be ignored. Moreover, Zhou et al. (2019) reported the use of PVA-alginate immobilized *G. oxydans* NL71 as a biocatalyst for whole-cell catalysis of xylose. Although the immobilized cells could improve the catalytic performance, with a volumetric yield of 7.10 g L^−1^ h^−1^ at a biomass concentration up to 10 g L^−1^, the *P*_*X*_ was only 0.71 g g_x_^−1^ h^−1^, which severely wasted the catalytic performance of *G. oxydans* NL71. The reason may be the oxygen transfer limitation imposed by immobilized cells, which limits the level of oxygen uptake.

**TABLE 5:** Comparative literature report on the production of XA under various process conditions.

References	Operating conditions	Strain	*C*_*X*_ (g L^−1^)	Xylose (g L^−1^)	*Qp* (g L^−1^ h^−1^)	*P*_*X*_ (g gx^−1^ h^−1^)
Present study	Agitation-728 rpm, aeration-7 L min^−1^	*G. oxydans* NL71	1.11	100	7.37	6.64
Zhang et al. (2017)	Agitation-500 rpm, aeration-2.5 L min^−1^	*G. oxydans*	2.50	63	5.42	2.17
		DSM 2003				
Zhou et al. (2015)	Agitation-500 rpm, aeration-3 L min^−1^	*G. oxydans* NL71	2	450	3.67	2.35
Zhou et al. (2017)	Agitation-500 rpm, aeration-3 L min^−1^	*G. oxydans* NL71	6	300	32.5	5.26
Dai et al. (2020)	Agitation-300 rpm, aeration not mentioned	*G. oxydans* NL71	4	141	4.48	1.12
Zhou et al. (2019)	Agitation-300 rpm, aeration- L min^−1^	*G. oxydans* NL71	10	200	7.10	0.71
Zhang et al. (2016b)	Agitation-500 rpm, aeration-2.5 L min^−1^	*G. oxydans* DSM 2003	1.5	40	1.43	-
Hou et al. (2018)	Agitation-500 rpm, aeration-1 vvm	*G. oxydans*	5%	54	0.82	-
		DSM 2003				
Hou et al. (2019)	Agitation-500 rpm, aeration-1 vvm	*G. oxydans*	5%	60	0.96	-
		DSM 2003				
Yim et al. (2017)	Shaking at 200 rpm	*Corynebacterium glutamicum*	2%	20	1.02	-
Liu et al. (2012)	Agitation-650 rpm, aeration-0.5 vvm	*E. coli*	3.2	40	1.09	0.34
Toivari et al. (2010a)	Agitation-500 rpm, aeration-01 vvm	*S. cerevisiae Xyd1*	4.6	20	0.03	0.0065
Toivari et al. (2010b)	Agitation-500 rpm, aeration-01 vvm	*S. cerevisiae* *B67002 xylB*	7	49	0.44	0.06

To solve the problem of low productivity caused by the mismatch between oxygen transfer and oxygen uptake, the fermentation conditions (agitation, aeration, biomass concentration) were optimized by Box-Behnken response surfaces. A final *P*_*X*_ of 6.64 ± 0.20 g gx^−1^ h^−1^ is achieved, which is significantly higher than that was reported in the literature. The above results indicate that the developed model can be used to improve the production efficiency of xylonic acid.

## Conclusion

The efficient bioconversion of xylonic acid using *G. oxydans* NL71 as a biocatalyst has been investigated using response surface methodology. Also, the maximum *P*_*X*_ for xylonic acid production was successfully obtained by multivariate optimization such as agitation, aeration and biomass concentration. A maximum specific productivity of 6.64 ± 0.20 g g_x_^−1^ h^−1^ for xylonic acid was obtained under optimized conditions (728 rpm, 7 L min^−1^, 1.11 g L^−1^). The optimized variables not only proved the validity of the models, but also effectively improved the production efficiency of *G. oxydans* NL71 in the whole-cell catalytic xylose process. Therefore, this study presents the potential application in improving the efficiency of xylonic acid production.

## Data Availability

The original contributions presented in the study are included in the article/supplementary material, further inquiries can be directed to the corresponding authors.
